# Adapting Sexual Behavior Survey Data to Parameterize an Agent-Based Model of Human Papillomavirus (HPV) Transmission

**DOI:** 10.1177/0272989X261425681

**Published:** 2026-03-06

**Authors:** Jennifer C. Spencer, Emily A. Burger, Allison Portnoy, Nicole G. Campos, Mary Caroline Regan, Stephen Sy, Jane J. Kim

**Affiliations:** Department of Population Health, Dell Medical School, UT Austin, Austin, TX, USA; Department of Internal Medicine, Dell Medical School, UT Austin, Austin, TX, USA; Department of Health Management and Health Economics, University of Oslo, Oslo, Blindern, Norway; Center for Health Decision Science, Harvard T.H. Chan School of Public Health, Boston, MA, USA; Department of Global Health, Boston University School of Public Health, Boston, MA, USA; Center for Health Decision Science, Harvard T.H. Chan School of Public Health, Boston, MA, USA; Center for Health Decision Science, Harvard T.H. Chan School of Public Health, Boston, MA, USA; Center for Health Decision Science, Harvard T.H. Chan School of Public Health, Boston, MA, USA; Center for Health Decision Science, Harvard T.H. Chan School of Public Health, Boston, MA, USA

**Keywords:** simulation model, sexually transmitted infection, human papillomavirus, calibration, vaccination

## Abstract

**Purpose:**

Sexual transmission of human papillomavirus (HPV) infection is important for capturing the indirect effects of interventions in mathematical models, but limited data create challenges for reflecting sexual behavior patterns over the lifespan of individuals and across heterogenous populations. We used nationally representative data from the United States to parameterize, calibrate, and validate a heterosexual transmission model of HPV.

**Methods:**

Based on sexual behavior data from the National Survey of Family Growth (2011–2019), we categorized respondents into 4 sexual activity categories, using their percentile of cumulative lifetime partners compared with others within their same sex and age group. We modeled probabilistic partnership acquisition and dissolution by age, sex, and sexual activity category. Partnership data were incorporated into an existing agent-based model of HPV transmission in the United States. We calibrated 1) per-partnership HPV transmission and 2) reduced risk of type-specific reinfection from natural immunity to fit age- and type-specific HPV prevalence using the National Health and Nutrition Examination Survey (NHANES 2002–2008). We validated the final model by comparing model-based projections of HPV prevalence against empirical data in the US population before and after widespread HPV vaccination.

**Results:**

After calibrating to fit overall HPV prevalence, model validation exercises indicated that the distribution of prevaccine HPV prevalence across sexual activity categories closely matched NHANES estimates. Simulating vaccination rates over 10 y, the model replicated postvaccine NHANES data for prevalence of HPV16.

**Conclusion:**

Capturing HPV transmission dynamics requires an understanding of sexual behavior across populations and over time. Defining sexual activity categories based on cumulative lifetime partners can capture patterns of HPV risk over a lifespan to reflect the dynamics of HPV transmission and vaccination.

**Highlights:**

- Using data from a large national survey, we developed sexual behavior inputs for an agent-based model of HPV transmission.
- We define 4 heterogenous risk groups using cumulative lifetime sexual behavior for males and females and find we can recreate validation data on both lifetime sexual patterns and age-specific HPV prevalence.
- Our calibrated model also reproduces early patterns of HPV reduction following HPV vaccine introduction.
- Modelers seeking to understand the long-term effects of the HPV vaccine should carefully consider the heterogeneity of sexual behavior across groups as well as changes in behavior over the lifespan.

## Graphical abstract

**Graphical abstract:**
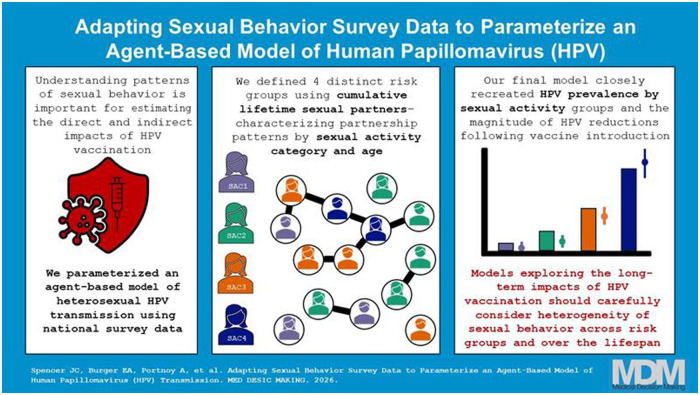

Persistent infection with high-risk human papillomavirus (HPV), a common sexually transmitted infection, causes nearly all cases of cervical cancer and a sizeable proportion of anal, oropharyngeal, vaginal, vulvar, and penile cancers. Prophylactic HPV vaccination can prevent acquisition of new infections.^1–3^ The HPV vaccine, first introduced in 2006, offers unprecedented potential to reduce the burden of HPV-associated cancers and is currently recommended by the Centers for Disease Control and Prevention’s Advisory Committee on Immunization Practices for individuals as early as age 9 y.^4^ Due to the long time horizon between preadolescent HPV vaccination, HPV infection after sexual initiation, and development of HPV-related cancers, simulation models have been crucial for shaping vaccination policy and understanding the implications of observed vaccination uptake patterns.^5–7^

To accurately project the health effects that include both direct and indirect (i.e., herd immunity) benefits from vaccination, mathematical simulation models have been developed using dynamic systems modeling or agent-based approaches, both of which explicitly capture interaction through sexual contacts and transmission of HPV.^6,8^ HPV is different from other sexually transmitted infections in several important ways.^9^ Evidence suggests that HPV is transmitted via skin-to-skin contact and thus may be transmitted during noninsertive sexual activity and despite condom use.^10,11^ HPV is highly prevalent, even among those with relatively few lifetime sexual partners,^12^ and because infection is largely asymptomatic, it can persist undetected for years or decades.^13,14^ Therefore, to understand the population-level impact of vaccination, it is important for HPV models to consider the wide heterogeneity of HPV exposure across age and sexual activity.

Existing dynamic HPV transmission models have used various methods to capture infection patterns across the US population and account for variation in sexual behavior within age cohorts and across the life course.^15–17^ Models of sexually transmitted infections generally reflect sexual behavior over the short term, using data on past-year partnerships to define a finite number of behavior categories.^18–20^ However, past-year partnerships may not accurately reflect accumulated lifetime risk. Whether and how much these identified sexual activity categories reflect distinct gradients of risk have not been well examined in simulation models. Incorporating differences in sexual exposure to HPV across populations and for individuals over their lifespan is important for capturing the cumulative risk of HPV infection, the timing and extent of herd immunity benefits, and the potential effects of a waning HPV vaccine.^6,21^ In this study, we developed and validated a new approach for parameterizing an agent-based model of HPV transmission using cumulative data on sexual behavior across the lifespan, derived from cross-sectional data on heterosexual sexual behavior in the United States.

## Methods

### Agent-Based Model Overview

We modified a previously developed agent-based model of HPV transmission in the United States, which simulates the formation and dissolution of heterosexual partnerships over the lifespans of multiple birth cohorts as well as the transmission of 7 independent high-risk HPV genotypes (HPV16, 18, 31, 33, 45, 52, and 58), which contribute to approximately 90% of cervical cancers and are targeted by the nonavalent vaccine.^22^ The agent-based model includes 4 primary components, which proceed in monthly time steps: 1) births and deaths, 2) sexual mixing, 3) HPV transmission, and 4) HPV clearance and natural immunity.

The agent-based model can be used in isolation or linked (using age-, genotype-, and birth cohort–specific HPV incidence) with a companion microsimulation model of the natural history of cervical carcinogenesis, enabling complex analyses of HPV vaccination and cervical cancer screening.^22,23^ Key updates from the previously published version of the model include recategorizations of sexual activity category (SAC), recalibration to HPV data from a national survey, and changing partnership duration parameters to instead reflect a monthly probability of separation (Supplemental Table 1).

### Model Parameterization and Assumptions

#### Sexual behavior inputs

We used data from the National Survey of Family Growth (NSFG), a population-based survey of the noninstitutionalized US population that assesses individual sexual behavior through an in-person computer-assisted survey, providing a high-quality estimation of heterosexual partnerships in 15- to 45-y-old males and females.^24^ We combined 4 cycles of data (2011–2013, 2013–2015, 2015–2017, 2017–2019) for a total sample size of 22,995 female and 19,067 male respondents. All analyses used provided survey weights to reflect the US population.

#### Sexual activity category

**Figure 1:**
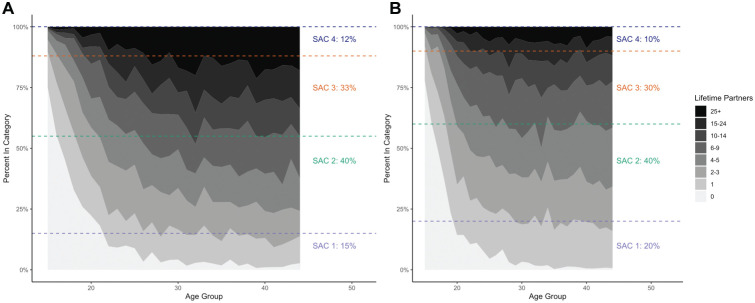
Definition of sexual activity category using cumulative lifetime partners, by sex: (A) males and (B) females.

To account for heterogeneity in sexual behavior and risk of HPV exposure, we categorized all respondents into 1 of 4 SAC groups. To approximate the trajectories of sexual behavior across the lifespan, we used the cumulative number of lifetime opposite-sex sexual partners by age (including reported number of partners for vaginal, oral, or anal sex) and visually divided respondents into 4 groups reflecting 1) low, 2) medium-low, 3) medium, and 4) high sexual activity. The lowest SAC group was defined in the data for both sexes as the portion reporting no partners or 1 lifetime partner (∼15% of males, 20% of females). The highest SAC group was identified for females as having 15 or more lifetime partners by age 30 y (top 10% of respondents) and for males as having 25 or more partners by age 30 y (top 12%). Finally, a cutoff was identified that created approximately evenly sized groups for the “medium-low” and “medium” groups. This means for males, we identified divides at the 0 to15th, 16th to 55th, 56th to 88th, and 89th to 100th percentiles. For female respondents, we identified these divides at the 0 to 20th, 21st to 60th, 61st to 90th, and 91st to 100th percentiles (Figure 1; Supplementary Table 1).

#### Sexual initiation and annual partnerships

Sexual behavior was modeled as individuals entering a mixing pool and “seeking” potential partnerships. The process of seeking partners and the number and type of partners sought in each month were based on data from NSFG. NSFG respondents were asked to report the age at which they first had sexual intercourse, from which we derived a cumulative probability of sexual behavior initiation by age and SAC (Supplementary Table 2). Among those who were sexually active, we calculated the mean number of opposite-sex sexual partners per year, by age and SAC. To reduce variation from year to year, we used a cubic function by age to smooth these estimates. We also identified an upper bound for annual partners by sex and age using the smoothed 75th percentile of past-year partners within each group (Supplementary Table 3). To create a distribution around these partnerships, we used the upper bound as the number of “potential” partnerships and calculated a probability modifier for males seeking a new partnership for each “potential” partnership (Supplementary Table 4). Sexual initiation data were retrospective and could start as early as 10 y of age. However, past-year partner data in NSFG did not begin until age 15 y; therefore, we estimated past-year partners for those younger than 15 y using cumulative lifetime partners by SAC at age 15 y as a guide. We further assumed the mean and maximum number of per-year partners stabilized starting at age 40 y and that separation rates continued to decline over time. We did not model any additional sexual behavior after 60 y of age.

#### Age and behavior assortativeness

Assortativity reflects the fact that individuals are more likely to form sexual partnerships with individuals close to their own age (age assortativity) or with similar sexual behavior patterns (SAC assortativity).^25^ Respondents were asked to report on the age of their most recent married or cohabiting partner. From this, we created age assortativity inputs that suggested males probabilistically selected female partners who were in the same 5-y age group (51.5%), in the next lowest 5-y age group (23.5%), in the next highest age group (13.6%), or in any other age group (9.6%). No data were available on partner selection by SAC; however, as separation within a partnership was dictated by male partners, to produce female relationship durations consistent with their SAC category (i.e., longer relationships for SAC 1 and short for SAC 4), we assumed that partnerships were generally assortative by SAC. We assumed males select partners most often from within the same SAC (65%), followed by the next lowest (30%), next highest (3%), or another (2%) SAC. If a nonexistent category was selected (for example, a male in SAC 1 trying to select a lower SAC), a partner from the same SAC was selected instead (Supplementary Table 5).

#### Partnership separation

NSFG respondents were asked to report detailed information on up to 3 past-year sexual partnerships (starting with their most recent sexual partner). For each, they were asked whether the relationship was still ongoing at the time of the survey. Annual age- and SAC-specific separation rates were defined for male respondents using the fraction of terminated partnerships in the past year. For SAC 1, a group defined by having only 1 cumulative lifetime sexual partner, we assumed partnerships were lifelong (Supplementary Table 6).

#### Nonsexual behavior inputs

Natural history of HPV was incorporated as previously described.^22^ Briefly, HPV transmission is simulated each month between each ongoing HPV-discordant partnership, based on calibrated sex- and type-specific transmission probabilities. Once acquired, an HPV infection could be cleared based on sex-, genotype-, and duration-specific probabilities.^22,26,27^ We assumed those who clear an HPV infection develop a degree of type-specific natural immunity, which partially protects against reinfection with the same HPV type.

### Model Calibration and Validation

#### HPV prevalence data

We derived HPV prevalence data from the National Health and Nutrition Examination Survey (NHANES), which is a nationally representative survey of the noninstitutionalized US population, which also collects detailed physical examination data and a variety of laboratory tests. Data on HPV prevalence were available for females aged 18 y and older. For model calibration, we used data from 3 cycles: 2003–2004, 2005–2006, and 2007–2008, which together included HPV data from 5,069 female respondents prior to national implementation of HPV vaccination in the United States. For validation of outcomes to post-HPV vaccination data, we used data from 2 cycles 2013–2014 and 2015–2016, which were the latest available data on HPV prevalence (data in 2017–2018 are not publicly available, and data collection in 2019–2020 was halted due to the COVID-19 pandemic). Together, these included HPV data from 3,843 female respondents. HPV prevalence data for males were derived from the HPV in Men (HIM) study, a prospective study of men recruited between 2005 and 2009.^28^ We used data from genital swabs for 1,309 US men to estimate the prevalence of HPV by genotype and age.

#### Model calibration

Model calibration aims to explore the plausible ranges of uncertainty in input parameters and identify values that, when combined within the model, best correspond with real-world data, known as calibration targets. Our 2-stage calibration first separately calibrated inputs related to each of the 7 included HPV types. For each, we calibrated the degree to which type-specific natural immunity following clearance of an infection reduced the probability of future type-specific reinfection for both males and females as well as the monthly probability of type-specific HPV transmission per infected-susceptible partnership (with separate inputs for male-to-female v. female-to-male transmission). We compared 5,000 combinations of each input varied across feasible ranges, consistent with best available data.^27,29–31^ We used a previously described likelihood-based approach to compare the projected outcomes from each set of input parameters against the relevant calibration targets.^32^ Calibration targets included age-, sex-, and genotype-specific HPV prevalence data prior to national implementation of HPV vaccination in the United States, according to NHANES and HIM. To account for uncertainty in the model parameters, we compared outcomes over a subset of 50 parameter sets that demonstrated a good correspondence with the empirical data. Calibrated models achieved a good fit to most prevalence targets, but model estimates were lower than expected across all HPV types for the youngest ages, despite our modeled sexual behavior meeting or exceeding the expected number of partners. Data from multiple surveys demonstrate distinct patterns of early-life sexual behavior, including a higher proportion of noninsertive sexual contact in those younger than 21 y.^33,34^ Therefore, we tested a series of transmission modifiers for those younger than age 21 y ranging from 1 to 2 based on approximations from NSFG data and found that 1.5 provided the best fit to NHANES data. We held this value constant across all HPV types.

#### Model validation: Sexual behavior and HPV prevalence by SAC

Given inconsistencies in self-reported sexual behavior by sex and age,^35^ cumulative lifetime behavior data may not be accurately reconstructed using cross-sectional data on past-year behavior. To assess the extent to which our model data recreated patterns of lifetime behavior from NSFG data, we compared model outputs to mean and median number of lifetime partners reported overall and by SAC.

We also assessed HPV16 prevalence in each of the 4 SAC categories in the model relative to the data. To recreate our SAC groups within NHANES data, we used the maximum number of lifetime sexual partners by age, which defined each SAC category from NSFG, and applied these categories to NHANES data on lifetime sexual partners. We then calculated the mean HPV16 prevalence for each group by age.

#### Model validation: Postvaccine HPV prevalence

To assess model validity to HPV prevalence following the introduction of HPV vaccination, we used NHANES data reflecting the period 7 to 10 y after HPV vaccine introduction (2013–2016) and compared HPV prevalence by age and genotype to model estimates using observed US HPV vaccination coverage data among adolescents and young adults for the first 10 y following introduction.^36,37^

## Results

### Calibration to HPV Prevalence

**Figure 2:**
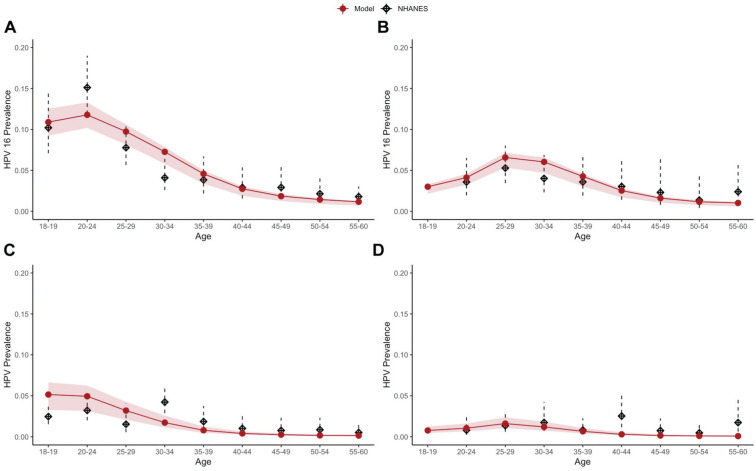
HPV16 and HPV18 prevalence in females (18–60 y): calibration and validation outcomes: (A) HPV16 (2003–2008), (B) HPV18 (2003–2008), (C) HPV16 (2013–2016), and (D) HPV18 (2013–2016). NHANES, National Health and Nutrition Examination Survey. NHANES data reflect survey-weighted means and the 95% confidence interval. Model output points reflect the best-fitting parameter set; the shaded area reflects the range of outcomes across the 50 top-fitting parameter sets.

After generating a repository of 5,000 unique input parameter sets, we retained the top 50 best-fitting sets. Calibrated parameters for genotype-specific natural immunity and monthly partnership transmission are shown in Supplemental Figure 1. All final parameter sets achieved acceptable visual fit to genotype-specific HPV prevalence parameters for females (Figure 2A and B; Supplementary Figure 2A–E) and for males (Supplementary Figure 3).

### Comparison to Sexual Behavior and HPV Data

**Figure 3:**
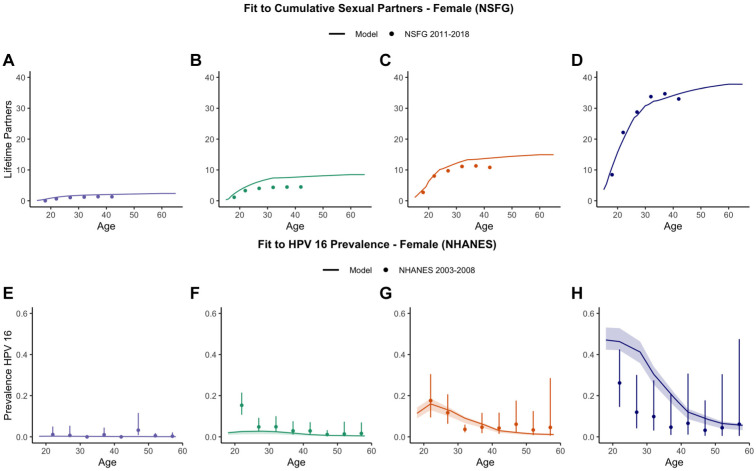
Model fit to cumulative sexual partners and HPV16 prevalence by sexual activity category—females: (A, E) SAC 1, (B, F) SAC 2, (C, G) SAC 3, and (D, H) SAC 4. Top: National Survey of Family Growth (NSFG). Points and lines show the mean from NSFG data. The confidence interval is not shown for NSFG data due to scale: confidence bounds average ±4.2% of the mean value. The solid lines show the model data for the mean number of lifetime partners by age among males. Bottom: National Health and Nutrition Examination Survey (NHANES). NHANES data reflect survey-weighted means and the 95% confidence interval. Model output points reflect the best-fitting parameter set, and the shaded area reflects the range of outcomes across the 50 top-fitting parameter sets.

Consistent with sex differences in partner reporting, the model projected lifetime sexual partners slightly higher than is reported in NSFG for females and slightly lower than is reported for males (Supplementary Figure 4). By age 40 y, women in the NSFG report an average of 8.5 partners, compared with our model estimate of 10.2 partners. By age 40 y, men in the NSFG report an average of 12 partners, compared with our model estimates of 10.5 lifetime partners. When comparing across SAC groups, we found our model generally replicated patterns of cumulative partners for both females (Figure 3A) and males (Supplementary Figure 5), with some underestimation of partners compared with NSFG in SAC 2 among females and for SAC 3 and 4 among males. When comparing the distribution of prevaccination HPV prevalence across our SAC groups, we found good fit to both the absolute and relative prevalence of HPV16 (Figure 3B), with the most variation at youngest age, for which we underestimated risk for SAC 2 relative to NHANES and overestimated risks for SAC 4 relative to NHANES.

### Validation to Postvaccination HPV

After simulating historical HPV vaccination uptake over time, we compared our model projections to data from NHANES following vaccination (2013 to 2016). We found that our model closely mirrored HPV16 and HPV18 prevalence by age (Figure 2C, D). Reductions in HPV prevalence were seen in only the youngest age groups, who would have been age-eligible for vaccination in the first 10 y of availability; minimal reductions were seen at older ages, consistent with the NHANES data. The prevalence of 5 non-HPV16/18 high-risk HPV genotypes, which were not included in the first available bivalent vaccine, showed minimal declines over time in both the model and NHANES data (Supplementary Figure 2F–J).

## Discussion

Our reparameterized model of heterosexual HPV transmission accurately reflected both population averages and heterogeneity in HPV risk by age, sex, and sexual behavior. Importantly, we were able to reflect differences in HPV prevalence across sexual behavior categories as well as reductions in HPV prevalence after the introduction of HPV vaccination that mirrored those in observational data from the US population. Using cumulative lifetime partners to generate sexual activity categories allowed us to replicate cumulative exposure to HPV and may be a better proxy for differentiating sexual risk groups than those using only past-year behavior.

Furthermore, we were able to reflect changes in behavior with age, including the increasing duration of relationships and slower acquisition of new partners. These patterns of behavior are essential for understanding the changing risk for HPV infection, as clinical studies are sparse regarding the population heterogeneity in the degree of natural immunity following clearance of HPV^13,38^ but suggest changing partnership patterns are the primary driver of HPV patterns by age. Accurately reflecting partnership change rates after age 30 y becomes particularly important when evaluating the impact of potential vaccine waning (e.g., with a single-dose regimen), where exposure patterns at these ages influence modeled risk and cancer outcomes.

High-quality data on sexual behavior can be difficult to collect. NSFG collects detailed data on multiple dimensions of past and current sexual behavior and uses a computer-assisted design in which interviewers lead questions but the respondents type their device into a computer (which the interviewer cannot see) to increase respondent privacy and improve the accuracy of reporting.^39,40^ NHANES also uses a computer-assisted approach for sexual behavior questions and has a high concordance with behaviors reported in NSFG.^41^ However, there are still known discrepancies between partnership reporting for males and females and differences in partnership reporting in the short term (past year) and long term (lifetime).^35,42^ We used repeat cross-sectional data across age groups to generate a pattern of partnership acquisition over time. Importantly, this could miss cohort effects, as norms around sexual behavior have shifted over time^42–44^ and we cannot separate the effect of age and birth cohort in the data. However, we found we are able to fit data on both cumulative behavior and HPV prevalence reasonably well across all age groups, suggesting a minimal impact of cohort effects.

This model captures only heterosexual transmission of HPV. An increasing number of young adults report gay, lesbian, bisexual, or queer sexual identities,^45^ making the dynamics of HPV across same-sex partnerships important to capture the full risk profile for HPV as well as the full impact of HPV vaccination. Further, our model captures transmission risk as a per-partnership per-month probability, which does not account for different transmission risks according to type of sexual activity (oral, anal, or vaginal sex) or for changes in the frequency of acts per month over time. To match the high prevalence of HPV at younger ages, we included a transmission multiplier at younger ages, which may help account for differences in behavior during this period, including potential transmission from noninsertive sexual partners, which is more common at younger ages.^19,33,46^ Future work should consider how exposures during this important period of sexual behavior may influence transmission patterns.

Finally, we acknowledge several limitations related to model inputs on the natural history of HPV. Our model assumes a type-specific reduction in the risk of HPV following clearance as a result of natural immunity, but we do not model potential reactivation of latent infections, which may account for a growing portion of newly detected HPV infections as age increases.^47,48^ Distinguishing a newly acquired infection from a reactivated latent one has potential implications, particularly for the impact of vaccination and screening in older women; our model could overestimate the benefits of vaccination and screening at older ages through assuming newly detected HPV infections are always the result of recent sexual activity.^49^ Data on reactivation are limited, as are data on the efficacy of HPV vaccination for reactivated infections (v. newly acquired infections). As new data are available, we will continue to update our model to reflect the best available information on HPV natural history.

We calibrated several key model inputs— including per-partnership transmission and natural immunity— which raises concerns of nonidentifiability (i.e., multiple values for these inputs could yield plausible model outputs, but we cannot be sure the calibration process has landed on the true values). To reduce the impact of a single parameter set, we repeated comparisons across the top 50 best-fitting sets and generally find consistency in model outputs. Numerous design aspects of our model, including the number of SACs and how data were divided to create these groups, are not deterministic and could be created in other ways. Approaches such as comparative modeling can be valuable for assessing the effect of these key structural decisions.^7,50^ We further note that a strength of our current approach is in validating that our division of these SAC groups reflects meaningful differences not only in behavior but also in HPV prevalence, which is replicable in our model.

Using high-quality, nationally representative data on sexual behavior in the United States, we derived parameters for an agent-based model of HPV transmission. We found that categorizing sexual behavior by cumulative lifetime partners created distinct risk groups and that short-term partnership data by age for each of these groups were sufficient to recreate observed data on lifetime partners and HPV risk. This approach allowed us to reflect not just population averages but distributions of HPV risk across age, sex, and sexual behavior groups. In the future, this validated model will be used to assess the effects of HPV vaccination in the United States and guide policy making in HPV vaccination and cancer prevention.

## Supplemental Material

sj-docx-1-mdm-10.1177_0272989X261425681 – Supplemental material for Adapting Sexual Behavior Survey Data to Parameterize an Agent-Based Model of Human Papillomavirus (HPV) Transmission Supplemental material, sj-docx-1-mdm-10.1177_0272989X261425681 for Adapting Sexual Behavior Survey Data to Parameterize an Agent-Based Model of Human Papillomavirus (HPV) Transmission by Jennifer C. Spencer, Emily A. Burger, Allison Portnoy, Nicole G. Campos, Mary Caroline Regan, Stephen Sy and Jane J. Kim in Medical Decision Making
