# Supplementary material for: Adapting Sexual Behavior Survey Data to Parameterize an Agent-Based Model of Human Papillomavirus (HPV) Transmission
Source: Med Decis Making. 2026 Mar 6;46(5):591–600. doi: 10.1177/0272989X261425681 (PMC12970602; doi:10.1177/0272989X261425681)
Supplement: sj-docx-1-mdm-10.1177_0272989X261425681 – Supplemental material for Adapting Sexual Behavior Survey Data to Parameterize an Agent-Based Model of Human Papillomavirus (HPV) Transmission [file sj-docx-1-mdm-10.1177_0272989X261425681.docx]

**Supplemental Figure 1: Distribution of Calibrated Model Inputs**

**
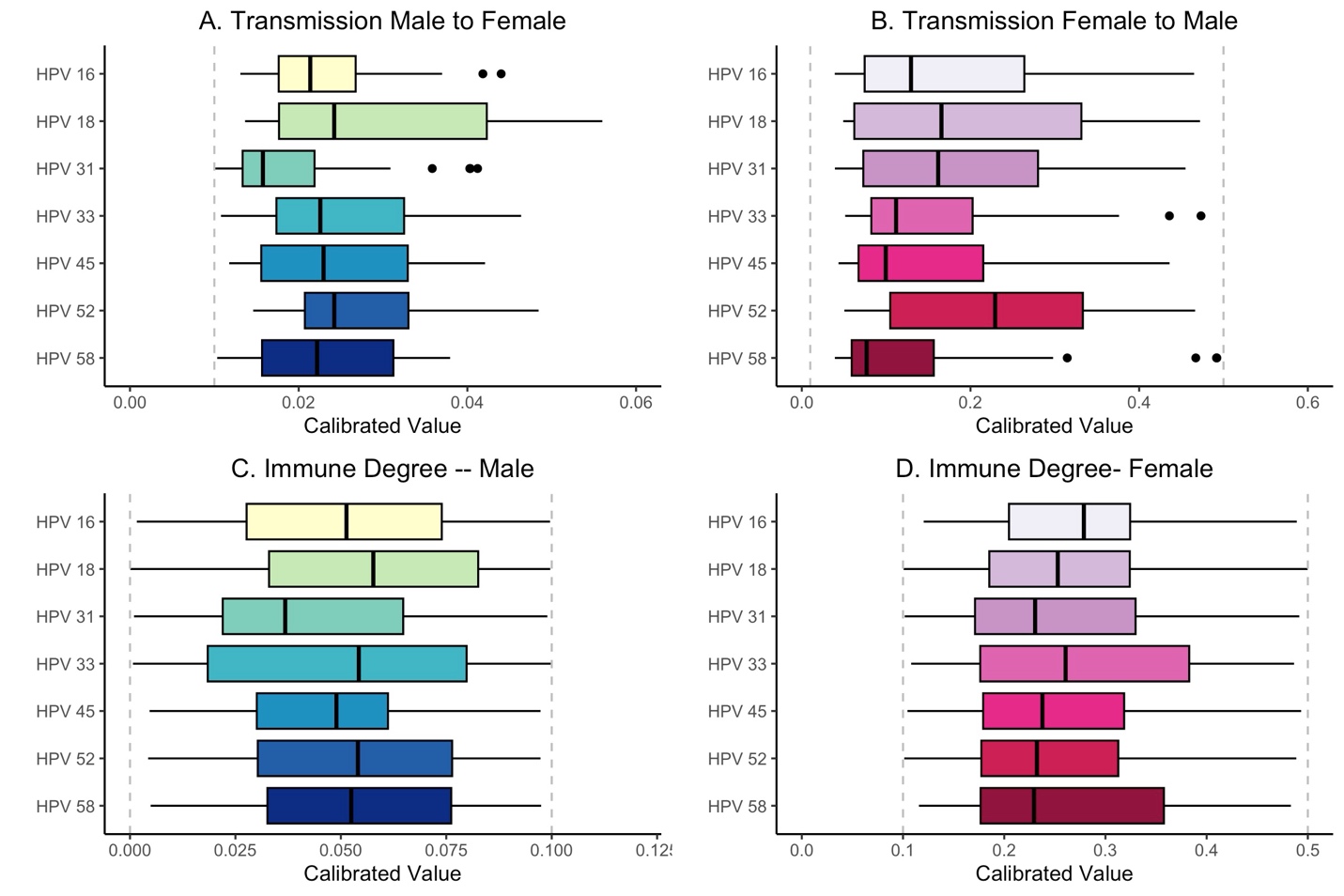
**

Boxplots show distribution of identified model inputs across calibration ranges for (a) transmission of HPV from infected male partner to uninfected female partner, (b) transmission of HPV from infected female partner to uninfected male partner, (c) the probability of natural immunity (i.e., reduction in risk of type-specific re-infection following clearance) of an HPV infection in males, and (d) the probability of natural immunity following clearance of an HPV infection in females. Dashed lines show upper and lower search ranges. The upper bound of the search range for male to female transmission was constrained by the selected female to male transmission parameter in each run.

**Supplemental Figure 2: Fit to HPV 31, 33, 45, 52, and 58 Pre-Vaccine (2003-2008) and Post-Vaccine (2013-2016)- Females (age 18-60)**


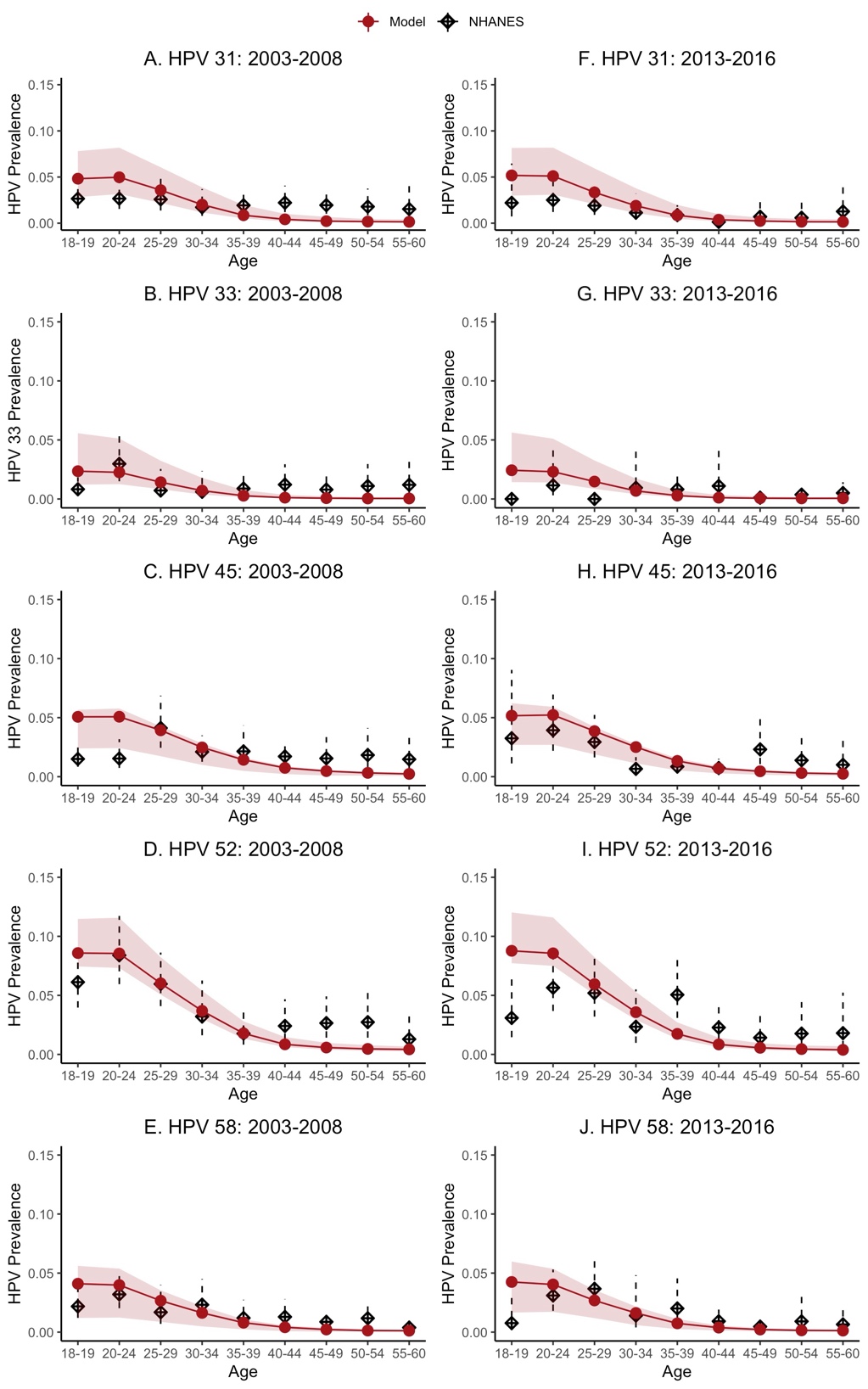


NHANES – National Health and Nutrition Examination Survey. NHANES data reflect survey-weighted means and 95% confidence interval. Model output points reflect best-fitting parameter set, shaded area reflects range of outcomes across the 50 top-fitting parameter sets.

**Supplemental Figure 4: Fit to HPV 16, 18, 31, 33, 45, 52, and 58 Pre-Vaccine (2005-2009) – Males (age 18-60)**


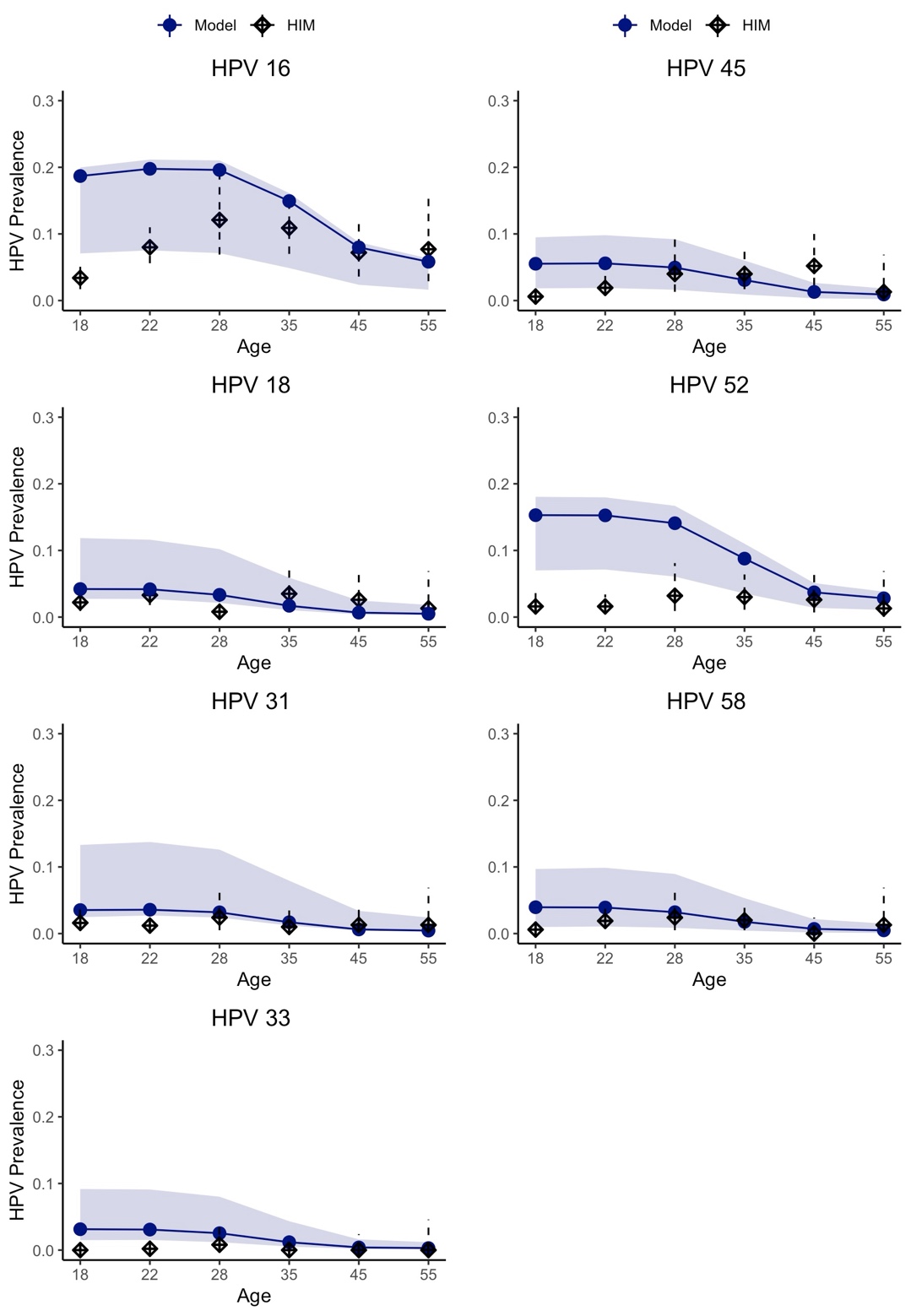


HIM – HPV in Men Study. HIM data reflects means and 95% confidence interval. Model output points reflect best-fitting parameter set, shaded area reflects range of outcomes across the 50 top-fitting parameter sets.

**Supplemental Figure 5: Model Fit to Cumulative Partners for Males and Females**

**
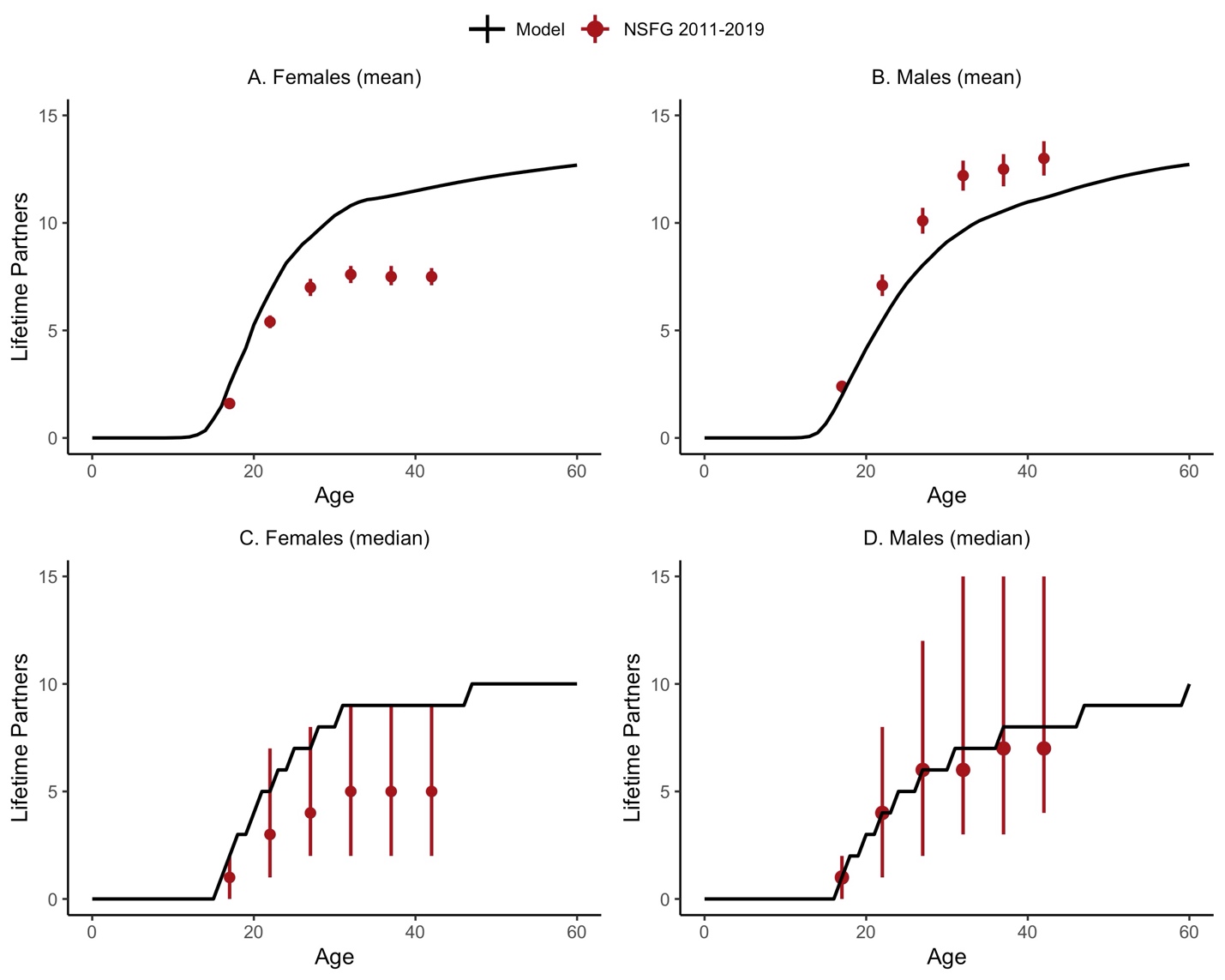
**

NSFG- National Survey of Family Growth. Red points and lines show mean and 95% confidence interval (Panels A & B) or median and interquartile range (Panels C & D). Black lines show model data for mean (Panels A & B) or median (Panels C & D).

**Supplemental Figure 6: Model Fit to Cumulative Sexual Partners and HPV 16 Prevalence by SAC in Males
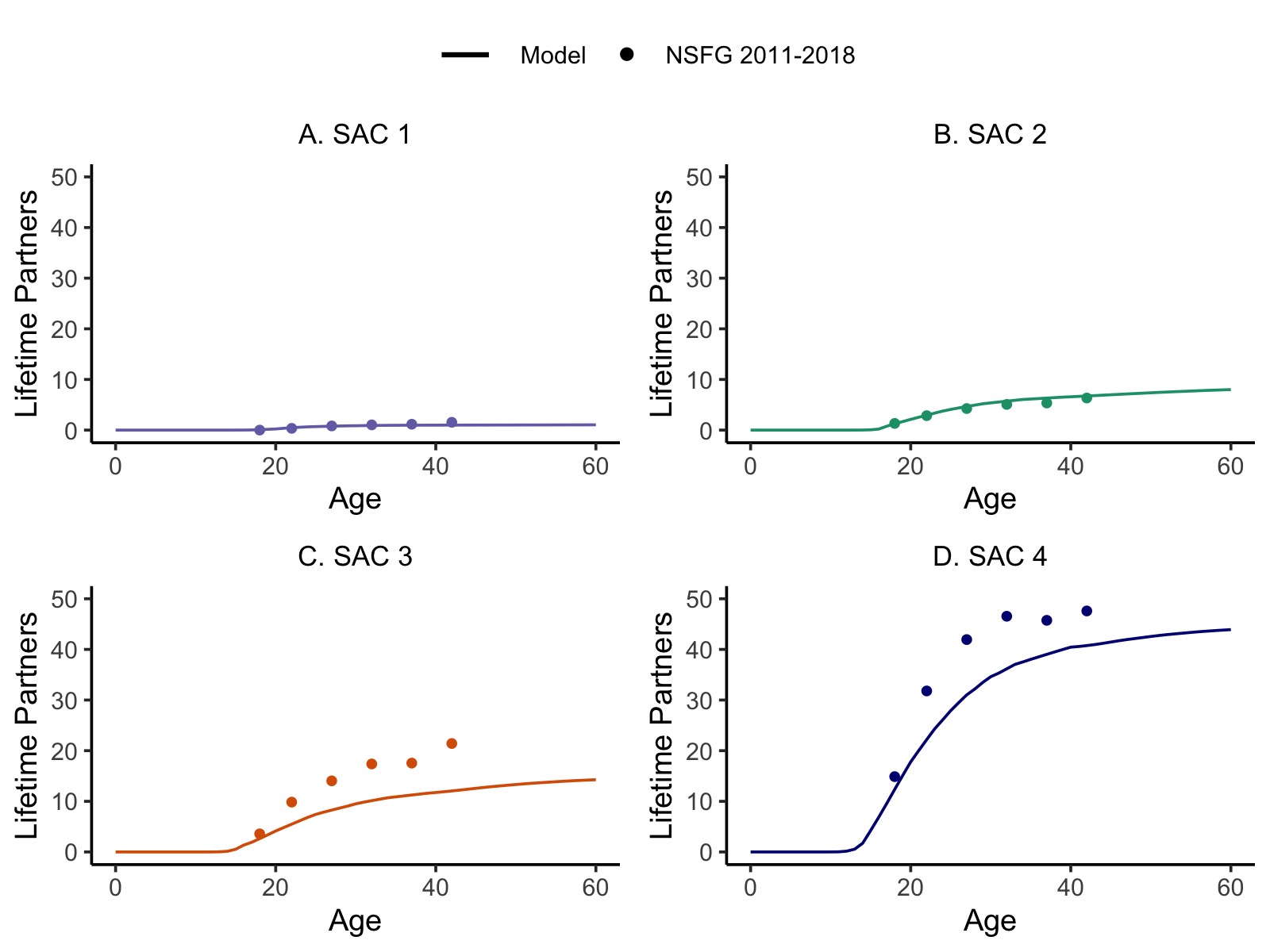
**

NSFG- National Survey of Family Growth. Points mean lifetime partners by age from NSFG data. Confidence interval not shown for NSFG data due to scale – confidence bounds average +/- 5.2% of mean value. Solid lines show model data for mean number of lifetime partners by age among males.

**Supplementary Table 1. Summary of Model Changes**

|  | Prior Model | New Model |
| --- | --- | --- |
| **Common Elements** | Agent-based model using monthly time-steps. Partnership formation is male-led and constrained by maximum number of partners, partnership probability modifier, and age- and SAC-specific assortativity | |
| **Changes** |  |  |
| SAC Groups | Defined by past year partnerships | Defined by cumulative lifetime partners |
| SAC Assortativity | Defined by two groups (same SAC or different SAC) | Defined by four groups (same SAC, higher SAC, lower SAC, other SAC) |
| Sexual debut | Implicit in partnership numbers | Explicit input – defined by reported age at first sex |
| Probability of partnership success | Calibrated to fit lifetime partnerships | Defined by past year partnerships |
| Partnership dissolution | Distribution of relationship duration at onset based on age and SAC | Monthly separation probability by age and SAC |
| Calibration Target Data | New Mexico HPV and Pap Registry | National Health and Nutrition Examination Survey |
| Calibrated Inputs | F to M ; M to F transmission; Immune Degree M, Immune Degree F (all type specific) | F to M ; M to F transmission; Natural immunity (all type specific) |

**S1. Sexual Activity Category Distribution by Sex**

|  | **Male** | **Female** |
| --- | --- | --- |
| SAC 1 | 15% | 20% |
| SAC 2 | 40% | 40% |
| SAC 3 | 33% | 30% |
| SAC 4 | 12% | 10% |

SAC, sexual activity category

**S2. Cumulative Probability of Sexual Initiation by Sex and Sexual Activity Category**

|  | **Male** | | | | **Female** | | | |
| --- | --- | --- | --- | --- | --- | --- | --- | --- |
| **Age** | **SAC1** | **SAC2** | **SAC3** | **SAC4** | **SAC1** | **SAC2** | **SAC3** | **SAC4** |
| 10 | 0% | 1% | 2% | 5% | 0% | 1% | 1% | 1% |
| 11 | 1% | 1% | 3% | 8% | 0% | 1% | 2% | 2% |
| 12 | 1% | 3% | 6% | 17% | 1% | 2% | 4% | 8% |
| 13 | 2% | 6% | 14% | 31% | 2% | 5% | 10% | 20% |
| 14 | 4% | 12% | 29% | 50% | 4% | 13% | 23% | 42% |
| 15 | 8% | 25% | 48% | 68% | 9% | 26% | 44% | 61% |
| 16 | 13% | 44% | 69% | 83% | 18% | 45% | 66% | 78% |
| 17 | 20% | 62% | 83% | 91% | 29% | 64% | 81% | 89% |
| 18 | 30% | 77% | 92% | 97% | 42% | 81% | 91% | 96% |
| 19 | 40% | 86% | 96% | 98% | 53% | 89% | 96% | 98% |
| 20 | 48% | 91% | 97% | 99% | 63% | 93% | 98% | 99% |
| 21 | 55% | 94% | 98% | 99% | 70% | 96% | 99% | 100% |
| 22 | 64% | 96% | 99% | 99% | 77% | 97% | 99% | 100% |
| 23 | 71% | 97% | 99% | 99% | 82% | 98% | 100% | 100% |
| 24 | 76% | 98% | 99% | 100% | 87% | 99% | 100% | 100% |
| 25 | 80% | 99% | 100% | 100% | 90% | 99% | 100% | 100% |
| 26 | 84% | 99% | 100% | 100% | 93% | 99% | 100% | 100% |
| 27 | 87% | 99% | 100% | 100% | 95% | 100% | 100% | 100% |
| 28 | 90% | 99% | 100% | 100% | 96% | 100% | 100% | 100% |
| 29 | 92% | 99% | 100% | 100% | 97% | 100% | 100% | 100% |
| 30 | 94% | 100% | 100% | 100% | 98% | 100% | 100% | 100% |

SAC, sexual activity category

**S3. Maximum Number of Per-Year Sexual Partners by Sex and Sexual Activity Category**

|  | **Male** | | | | **Female** | | | |
| --- | --- | --- | --- | --- | --- | --- | --- | --- |
| **Age** | **SAC1** | **SAC2** | **SAC3** | **SAC4** | **SAC1** | **SAC2** | **SAC3** | **SAC4** |
| 10 | 0* | 1* | 1* | 3* | 0* | 1* | 1* | 1* |
| 11 | 0* | 1* | 1* | 4* | 0* | 1* | 1* | 1* |
| 12 | 0* | 1* | 1* | 5* | 0* | 1* | 1* | 2* |
| 13 | 0* | 1* | 2* | 5* | 0* | 1* | 1* | 3* |
| 14 | 0* | 1* | 2* | 6* | 0* | 1* | 1* | 3* |
| 15 | 1* | 1 | 2 | 6 | 1* | 1 | 2 | 4 |
| 16 | 1* | 1 | 2 | 7 | 1* | 1 | 2 | 4 |
| 17 | 1* | 2 | 2 | 7 | 1* | 2 | 2 | 5 |
| 18 | 1 | 2 | 2 | 7 | 1 | 2 | 2 | 5 |
| 19 | 1 | 2 | 2 | 7 | 1 | 2 | 2 | 5 |
| 20 | 1 | 2 | 2 | 7 | 1 | 2 | 3 | 5 |
| 21 | 1 | 2 | 2 | 7 | 1 | 2 | 3 | 5 |
| 22 | 1 | 2 | 2 | 7 | 1 | 2 | 3 | 5 |
| 23 | 1 | 2 | 2 | 7 | 1 | 2 | 3 | 5 |
| 24 | 1 | 2 | 2 | 6 | 1 | 2 | 3 | 5 |
| 25 | 1 | 2 | 2 | 6 | 1 | 2 | 2 | 5 |
| 26 | 1 | 2 | 2 | 6 | 1 | 2 | 2 | 5 |
| 27 | 1 | 2 | 2 | 6 | 1 | 2 | 2 | 4 |
| 28 | 1 | 2 | 2 | 5 | 1 | 2 | 2 | 4 |
| 29 | 1 | 2 | 2 | 5 | 1 | 2 | 2 | 4 |
| 30 | 1 | 2 | 2 | 5 | 1 | 2 | 2 | 4 |
| 31 | 1 | 2 | 2 | 4 | 1 | 2 | 2 | 3 |
| 32 | 1 | 2 | 2 | 4 | 1 | 2 | 2 | 3 |
| 33 | 1 | 2 | 2 | 4 | 1 | 1 | 2 | 3 |
| 34 | 1 | 2 | 2 | 3 | 1 | 1 | 1 | 2 |
| 35 | 1 | 2 | 2 | 3 | 1 | 1 | 1 | 2 |
| 36 | 1 | 2 | 2 | 3 | 1 | 1 | 1 | 2 |
| 37 | 1 | 2 | 2 | 3 | 1 | 1 | 1 | 2 |
| 38 | 1 | 2 | 2 | 3 | 1 | 1 | 1 | 2 |
| 39 | 1 | 2 | 2 | 3 | 1 | 1 | 1 | 2 |
| 40 | 1 | 2 | 2 | 3 | 1 | 1 | 1 | 2 |
| 41-60 | 1** | 2** | 2** | 2** | 1** | 1** | 1** | 2** |

SAC, sexual activity category. *Number of past year partners was not collected before age 15, numbers were extrapolated from total partners at age 15

**Data on past year partners was not collected after 45 years of age. We assumed partnership numbers stabilized after age 40.

**S4. Annual Partnership Probability Modifier for Males by Sexual Activity Category**

| **Age** | **SAC1** | **SAC2** | **SAC3** | **SAC4** |
| --- | --- | --- | --- | --- |
| 10 | 0* | 0.01* | 0.01* | 0.05* |
| 11 | 0* | 0.01* | 0.03* | 0.08* |
| 12 | 0* | 0.03* | 0.06* | 0.17* |
| 13 | 0* | 0.06* | 0.14* | 0.31* |
| 14 | 0* | 0.12* | 0.29* | 0.50* |
| 15 | 0.08 | 0.25 | 0.48 | 0.80 |
| 16 | 0.13 | 0.43 | 0.8 | 0.69 |
| 17 | 0.20 | 0.62 | 0.59 | 0.69 |
| 18 | 0.51 | 0.62 | 0.63 | 0.68 |
| 19 | 0.49 | 0.51 | 0.67 | 0.67 |
| 20 | 0.46 | 0.52 | 0.69 | 0.66 |
| 21 | 0.89 | 0.53 | 0.71 | 0.65 |
| 22 | 0.88 | 0.54 | 0.72 | 0.64 |
| 23 | 0.86 | 0.55 | 0.72 | 0.62 |
| 24 | 0.86 | 0.55 | 0.71 | 0.70 |
| 25 | 0.85 | 0.56 | 0.70 | 0.68 |
| 26 | 0.85 | 0.56 | 0.69 | 0.65 |
| 27 | 0.85 | 0.55 | 0.67 | 0.63 |
| 28 | 0.86 | 0.55 | 0.65 | 0.72 |
| 29 | 0.87 | 0.55 | 0.62 | 0.69 |
| 30 | 0.87 | 0.54 | 0.89 | 0.65 |
| 31 | 0.88 | 0.54 | 0.85 | 0.78 |
| 32 | 0.89 | 0.53 | 0.81 | 0.74 |
| 33 | 0.90 | 0.52 | 0.77 | 0.70 |
| 34 | 0.91 | 0.52 | 0.73 | 0.88 |
| 35 | 0.92 | 0.51 | 0.69 | 0.84 |
| 36 | 0.93 | 0.51 | 0.66 | 0.79 |
| 37 | 0.93 | 0.50 | 0.63 | 0.75 |
| 38 | 0.93 | 0.50 | 0.61 | 0.72 |
| 39 | 0.93 | 0.50 | 0.60 | 0.69 |
| 40 | 0.93 | 0.50 | 0.60 | 0.69 |
| 41 | 0.92 | 0.50** | 0.60** | 0.69** |
| 42 | 0.91 | 0.50** | 0.62** | 0.69** |
| 43 | 0.89 | 0.51** | 0.65** | 0.64** |
| 44-60 | 0.87** | 0.52** | 0.69** | 0.64** |

SAC, sexual activity category. *Number of past year partners was not collected before age 15, numbers were extrapolated from total partners at age 15 **Data on past year partners was not collected after 45 years of age. We assumed partnership numbers stabilized after age 40.

**S5: Assortativity Parameters***

|  | **Male** |
| --- | --- |
| **Age:** |  |
| F partner same age group | 51.5% |
| F partner 1 group younger | 25.3% |
| F partner 1 group older | 13.6% |
| F partner any other age group | 9.6% |
| **SAC:** |  |
| F partner same SAC | 65% |
| F partner 1 SAC lower | 30% |
| F partner 1 SAC higher | 3% |
| F partner any other SAC | 2% |

SAC, sexual activity category.

**S6: Separation Probability Monthly (Male Partner) by Sexual Activity Category**

| **Age** | **SAC1** | **SAC2** | **SAC3** | **SAC4** |
| --- | --- | --- | --- | --- |
| 10 | 0 | 0.035969* | 0.067367* | 0.077734* |
| 11 | 0 | 0.035969* | 0.067367* | 0.077734* |
| 12 | 0 | 0.035969* | 0.067367* | 0.077734* |
| 13 | 0 | 0.035969* | 0.067367* | 0.077734* |
| 14 | 0 | 0.035969* | 0.067367* | 0.077734* |
| 15 | 0 | 0.035969 | 0.067367 | 0.077734 |
| 16 | 0 | 0. 035969 | 0.067367 | 0.077734 |
| 17 | 0 | 0. 035969 | 0.067367 | 0.077734 |
| 18 | 0 | 0. 035969 | 0.067367 | 0.077734 |
| 19 | 0 | 0. 035969 | 0.067367 | 0.077734 |
| 20 | 0 | 0.028024 | 0.050232 | 0.050184 |
| 21 | 0 | 0.028024 | 0.050232 | 0.050184 |
| 22 | 0 | 0.028024 | 0.050232 | 0.050184 |
| 23 | 0 | 0.028024 | 0.050232 | 0.050184 |
| 24 | 0 | 0.028024 | 0.050232 | 0.050184 |
| 25 | 0 | 0.016225 | 0.025117 | 0.037570 |
| 26 | 0 | 0.016225 | 0.025117 | 0.037570 |
| 27 | 0 | 0.016225 | 0.025117 | 0.037570 |
| 28 | 0 | 0.016225 | 0.025117 | 0.037570 |
| 29 | 0 | 0.016225 | 0.025117 | 0.037570 |
| 30 | 0 | 0.009667 | 0.016639 | 0.029587 |
| 31 | 0 | 0.009667 | 0.016639 | 0.029587 |
| 32 | 0 | 0.009667 | 0.016639 | 0.029587 |
| 33 | 0 | 0.009667 | 0.016639 | 0.029587 |
| 34 | 0 | 0.009667 | 0.016639 | 0.029587 |
| 35 | 0 | 0.005953 | 0.011339 | 0.020582 |
| 36 | 0 | 0.005953 | 0.011339 | 0.020582 |
| 37 | 0 | 0.005953 | 0.011339 | 0.020582 |
| 38 | 0 | 0.005953 | 0.011339 | 0.020582 |
| 39 | 0 | 0.005953 | 0.011339 | 0.020582 |
| 40-60 | 0 | 0.005746** | 0.010100** | 0.016401** |

SAC, sexual activity category.

*Number of partnership separation was not collected before age 15, numbers were assumed the same as data for age 15

**Data on partnership separation was not collected after 45 years of age. We assumed partnership numbers decreased at age 40 and stabilized thereafter.
